# The expression of HSP27 is associated with poor clinical outcome in intrahepatic cholangiocarcinoma

**DOI:** 10.1186/1471-2407-7-232

**Published:** 2007-12-21

**Authors:** Antonello A Romani, Pellegrino Crafa, Silvia Desenzani, Gallia Graiani, Costanza Lagrasta, Mario Sianesi, Paolo Soliani, Angelo F Borghetti

**Affiliations:** 1 Dipartimento di Medicina Sperimentale – Sezione di Patologia Molecolare ed Immunologia, Università degli Studi di Parma, 43100 Parma, Italy; 2 Dipartimento di Patologia e Medicina di Laboratorio – Sezione di Anatomia ed Istologia Patologica, Università degli Studi di Parma, 43100 Parma, Italy; 3 Dipartimento di Scienze Chirurgiche – Sezione di Clinica Chirurgica Generale e dei Trapianti d'Organo, Università degli Studi di Parma, 43100 Parma, Italy

## Abstract

**Background:**

The heat shock proteins (HSPs) 27-kDa (HSP27) and 72-kDa (HSP72), are ubiquitous chaperone molecules inducible in cells exposed to different stress conditions. Increased level of HSPs are reported in several human cancers, and found to be associated with the resistance to some anticancer treatments and poor prognosis. However, there is no study of the relationship between HSPs expression and patient's prognosis in intrahepatic cholangiocarcinoma (IHCCA). In this exploratory retrospective study, we investigated the expressions of HSP27 and HSP72 as potential prognostic factors in IHCCA.

**Methods:**

Thirty-one paraffin-embedded samples were analyzed by immunohistochemical methods using HSP27 and HSP72 monoclonal antibodies. Proliferation rate was assessed in the same specimens by using monoclonal antibody against phosphorylated histone H3 (pHH3). Fisher's exact test was used to assess the hypothesis of independence between categorical variables in 2 × 2 tables. The ANOVA procedure was used to evaluate the association between ordinal and categorical variables. Estimates of the survival probability were calculated using the Kaplan-Meier method, and the log rank test was employed to test the null hypothesis of equality in overall survival among groups. The hazard ratio associated with HSP27 and HSP72 expression was estimated by Cox hazard-proportional regression.

**Results:**

The expression of HSP27 was related to mitotic index, tumor greatest dimension, capsular and vascular invasion while the expression of HSP72 was only related to the presence of necrosis and the lymphoid infiltration. Kaplan-Maier analysis suggested that the expression of HSP27 significantly worsened the patients' median overall survival (11 ± 3.18 vs 55 ± 4.1 months, P-value = 0.0003). Moreover HSP27-positive patients exhibited the worst mean survival (7.0 ± 3.2 months) in the absence of concomitant HSP72 expression.

**Conclusion:**

The expression of HSP27, likely increasing cell proliferation, tumor mass, vascular and capsular invasion, might promote aggressive tumor behaviour in IHCCA and decrease patients' survival. Immunohistochemical detection of HSP27 on routine sections may provide a reliable prognostic marker for IHCCA able to influence the therapeutic strategies for this cancer.

## Background

Cholangiocarcinoma is a malignant epithelial tumor derived from the bile duct epithelium and the second most common primary hepatobiliary cancer, after hepatocellular carcinoma[1]. Cholangiocarcinoma remains a relatively rare disease, accounting for <2% of all human malignancies [2]. Despite aggressive screening, most patients thought to have localized disease at diagnosis present with advanced stage tumor not amenable to surgical treatment. Chemotherapy and radiotherapy are ineffective in patients with inoperable tumors, and biliary drainage is the mainstay of palliation[3]. The median survival time for patients with intrahepatic cholangiocarcinoma without hilar involvement varies from 18–30 months.

HSPs are one of the most evolutionarily conserved classes of molecules and play a fundamental role in the maintenance of cellular homeostasis. Under physiological conditions, they act as molecular chaperones, by assisting protein folding, oligomerization and translocation[4]. During stress they are overexpressed, preventing aggregation and promoting refolding of damaged proteins. Apoptosis resistance is often associated with high expression of heat-shock proteins [5-7] (HSPs), and various HSPs inhibitors can induce apoptosis in many tumor models [8]. These mechanisms underscore the role of HSPs in tumor progression and resistance to treatment [9]. The mechanism of HSPs overexpression in cancer is still a matter of debate [10]. The physiopathological features of the tumor microenvironment such as low glucose, low pH and low oxygen are likely to be involved.

In spite of these observations, attempts to correlate the levels of HSPs in tumor samples with clinical prognosis and progression in man have provided controversial results.

We focused our attention on intrahepatic cholangiocarcinoma (IHCCA), prompted by epidemiological data, showing a marked increase incidence in our province as well as in the most Western countries[11], and the absence of previous investigations. Aim of the study was the assessment of tissue expression of HSP72 and HSP27 in IHCCA resected with curative intention and the correlation with patients survival.

## Methods

### Patients

Epidemiological data, from the Parma Tumor Registry (Divisione di Oncologia Medica, Azienda Ospedaliera di Parma), indicated a marked increase in the incidence of IHCCA in our province from 1–2 cases/year to 9–12 cases/year over the period 1994–2004. Base of the study was a group of 31 patients with IHCCA who underwent resection with radical intent (R0) at the Dipartimento di Scienze Chirurgiche, Sezione di Clinica Chirurgica Generale e dei Trapianti d'Organo, over the period 1998–2006.

Background information including clinical-pathologic data and patients' survival was retrieved from histological and clinical records. None of the patients had received chemotherapy or radiotherapy prior to surgery.

The study protocol was in accordance with the recommendation of the Declaration of Helsinki. and the indications of Italian DLgs no. 196/03 (Codex on Privacy). Written consent to use stored tissue was obtained from all living patients.

### Sample sources

All pathological samples were retrieved from the archives of the Dipartimento di Patologia e Medicina di Laboratorio – Sezione di Anatomia ed Istologia Patologica. Formalin-fixed, paraffin-embedded, hematoxylin-eosin-stained sections were reviewed and re-staged according to the recent AJCC classification[12]. Representative areas of the tumors, adjacent normal and dysplastic liver were selected for immunohistochemistry studies.

### Immunohistochemistry

Immunohistochemistry was performed as previously described[13]. The paraffin sections were dewaxed, rehydrated, washed in Tris-buffered saline (TBS) (150 mmol/L NaCl, 50 mmol/L Tris, pH 7.4). The antigen determinants masked by formalin-fixation and paraffin-embedding were exposed by heating the section in a microwave oven for 10 min in an *epitope retrieval solution *(10 mM Tris Base, 1 mM EDTA solution, 0.05% (v/v) Tween 20, pH 9.0). The endogenous peroxidase activity was quenched with 3% H_2_O_2 _in deionized water for 10 min. Non-specific bindings were blocked in TBS containing 5% fraction V bovine albumin (Sigma-Aldrich, St. Louis, MO, USA), 5% normal mouse serum (Dako Corp., Carpinteria, CA) and 0.1% porcine gelatin type B (Sigma-Aldrich). Primary monoclonal antibodies against HSP27, clone G3.1, and inducible HSP72, clone C92F3A-5 (StressGene, Victoria, BC Canada), and phosphorylated histone H3 (pHH3), clone 8656-R (Santa Cruz Biotechnology, Inc. Santa Cruz, CA), were used at 1:200, 1:600 and 1:50 dilutions, respectively. All samples were incubated for 60 min with primary antibodies at room temperature. Immunoperoxidase staining was performed using an anti-Mouse/Rabbit Poly HRP Detection Kit (Chemicon International, Temecula, CA) according to the manufacture's specifications and developed with DAB. The specimens were counterstained with hematoxylin, mounted and examined by light microscopy. Routine negative controls used TBS instead of the primary antibody. An isotype control was conducted using a mouse IgG isotype control serum (Dako Corp., Carpinteria, CA). Negative and isotype controls were used in all staining runs. All negative and isotype controls resulted in negative immunohistochemical reactions.

The staining intensities of the IHCCAs were compared with those of adjacent non-neoplastic biliary ducts by visual evaluation by three observers. Overexpression was considered to be present when the intensity of tumor staining was at least twice the staining intensity of adjacent non-neoplastic areas.

### Mitotic count

Mitotic index of cancer cells was assessed by pHH3 staining, provide a simple and a reliable method for quantifying proliferative potential and an indipendent predictor of survival [14].

### Image Analysis

The expression of HSP27 or HSP72 was evaluated using a microscope image analysis system (Nikon Digital Net system). For each patient, five randomized, non-overlapping frames were evaluated in the same neoplastic area at 400× field magnification (0.198 mm^2^·field). At least 50 positive cells were counted for a total of 250–500 cells analyzed. To assess reproducibility of the image analysis, the pathologist blindly scored the 66% of the total of patients and also randomly selected frames. Although agreement was not exact in every case, no significant variation between the investigators' analyses (PC, AAR, SD) was noted.

### Statistical analysis

The biomarker and clinical data were analyzed using S-Plus Professional Edition v.6.1 (Insightful Corp., Seattle, WA). Fisher's Exact Test was used to test the hypothesis of independence between categorical variables in 2 × 2 tables. The ANOVA procedure was used to evaluate the association between ordinal and categorical variables. All tests were two-sided. The conversion of a continuous covariate into a binary one when there is no established cut-off point (previous published results or biological knowledge) was performed by using an outcome-oriented statistical method (such as the optimal cutpoint estimation). For optimal cutpoint estimation a corrected P value method, as described by Hothorn and Lausen, was used. Estimates of the survival probability were calculated using the Kaplan-Meier method, and the log rank test was employed to test the null hypothesis of equality in overall survival among groups. The hazard ratio associated with HSP27 and HSP72 expression was estimated by Cox hazard-proportional regression.

## Results

The study considered a eight year-period of inclusion, with a satisfactory homogeneity of patients treated with radical intent. The average age of the 20 females and 11 males undergoing surgery for IHCCA was 68 years (range 52–79). Two patients were positive to C viral hepatitis (one of them showed B and C coinfection). None of the other patients had underlying chronic liver disease. At presentation, twelve patients had one comorbidity (diabetes, hypertension or heart disease), eight patients had two comorbidities (mainly diabetes and hypertension), and only two patients had three comorbidities (chronic obstructive lung disease, diabetes and heart disease).

The surgical procedures, included: 8 right trisectionectomies, 6 right and 4 left hemihepatectomies (including the extended), 9 sectoriectomies (anterior, posterior and lateral) and 4 segmentectomies. The mean liver volume removed was 49 ± 19.2% (range 22–75%) while 4.6 ± 2.1 were the mean number of resected hepatic segments.

Twenty-four (77%) out of 31 tumors were well to moderately differentiated, and 7 (23%) poorly differentiated. The tumor growth pattern was papillary in eight cases, glandular in ten, and solid in thirteen. Patient characteristics are summarized in Table 1.

**Table 1:** Clinical-pathological features of patients affected by IHCCA who underwent surgery

*Characteristics*	Total patients (N = 31)
*Age at surgery*	67.7 ± 7 yrs
*Gender*	
Male	11
Female	20
*Tumor greatest dimension*^#^	
<4 cm	8
4–6 cm	9
>6 cm	10
*Histologic grade*^1#^	
Well/Moderate	20
Poor	8
*Presence of tumor necrosis*^1#^	
<50%	9
>50%	22
*Peritumoral border liver*^1#^	
Infiltrated	12
Pushed	18
*Lymphoid infiltration*^1#^	
Absent	18
Present	10
*Stage*^#^	
I	6
II	5
III	17

^1^Estimated by pathologist; ^#^data were not available for some patients.

None of the 31 liver samples exhibited HSP27 or HSP72 expression in normal biliary tract epithelium, or in dysplastic areas. A low positivity was only observed in hepatocytes lining tumor invasion border (not shown). This low positivity could be related to the stress determined by increasing mechanic pressure of the enlarging tumor mass and similar to the stress response induced by high hydrostatic pressure in many types of mammalian cells[15].

**Figure 1:**
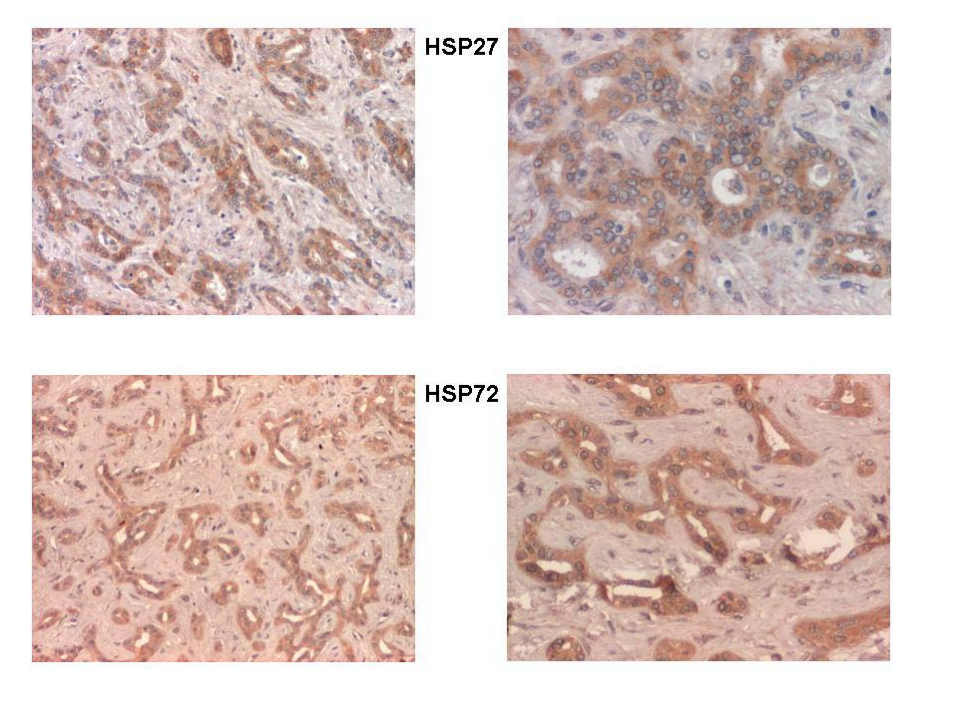
**Immunoperoxidase staining**. Serial paraffin-embedded tissue sections of IHCCA show HSP27 and HSP72 expression in cholangiocarcinoma cells (brown staining). Left panels, 100× magnification; right panels, 200× magnification.

Ten tumors samples (32%) were immunonegative for HSPs, whereas 21 (68%) showed detectable immunoreactivity for HSP27 or/and HSP72 (Figure 1). A single HSP27 or HSP72 immunopositivity was found in 6 and 7 samples, respectively, while in 8 samples concomitant expression of HSP27 and HSP72 was observed.

**Figure 2:**
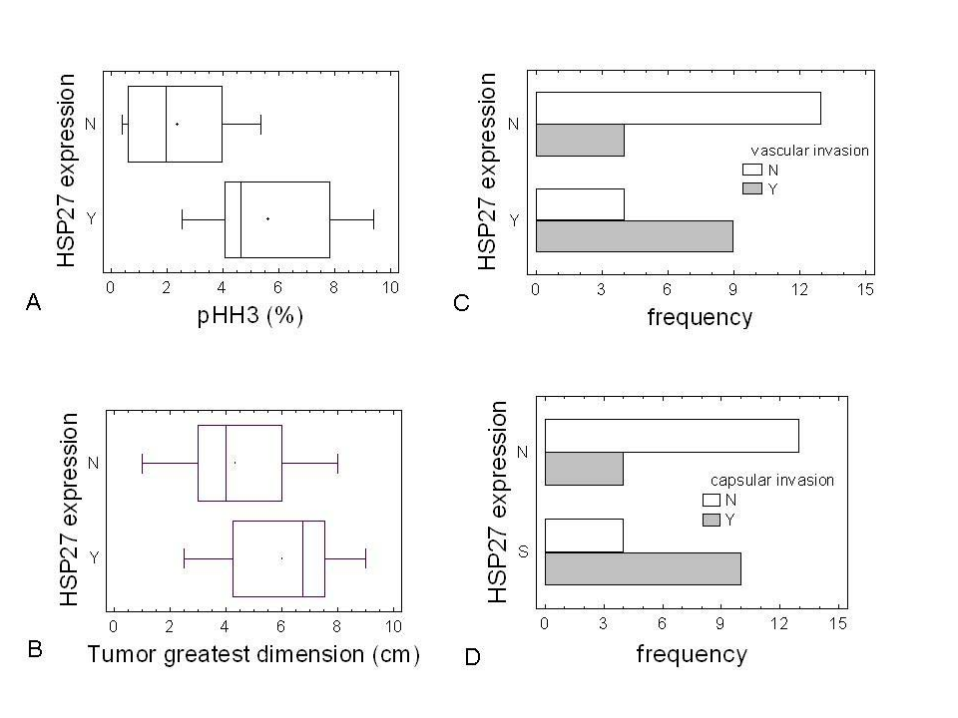
**HSP27 expression versus tumor parameters**. *Box-and-whisker *plots of HSP27 expression versus percentage of pHH3-positive cells (A), and of HSP27 versus tumor greatest dimension (B). The open squares represent those outlier patients outside the 95% confidence interval. The black solid circles represent the mean values, while the line inside the box represents the median. *Bar-chart *plots of HSP27 expression versus frequency of vascular (C) and capsular (D) invasion.

**Figure 3:**
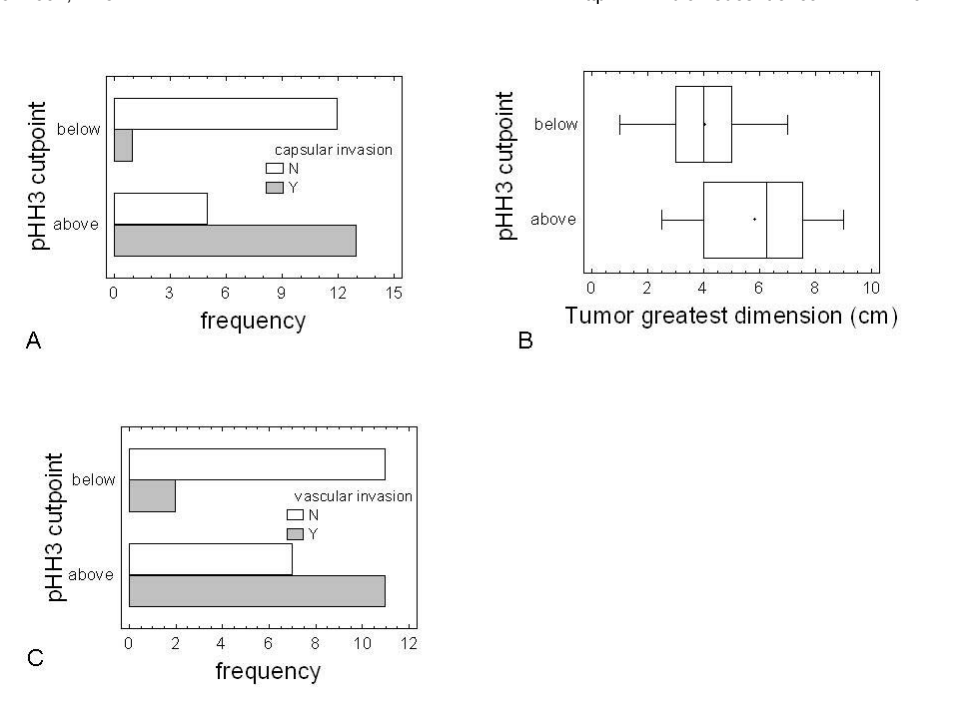
**pHH3 cutpoint versus tumor progression parameters**. *Bar-chart *plots of pHH3 below/above cutpoint versus frequency of vascular (A) and capsular (C) invasion. *Box-and-whisker *plots of pHH3 cutpoint versus tumor greatest dimension (B). The black solid circles represent the mean values, while the line inside the box represents the median.

HSP27 expression was significantly associated with cell proliferation as assessed by pHH3 staining (P-value = 0.0015) (Figure 2A), and was positively correlated with main clinic-pathological features of tumor: its expression showed a marginal significant (P-value = 0.047) correlation to the greatest tumor dimension (4.36 ± 0.51 vs 6.05 ± 0.57 cm) (Figure 2B). A significant correlation (P-value = 0.004) between HSP27 and tumor vascular invasion was also observed (Figure 2C). Both HSP27 expression (Figure 2D) and pHH3 count (Figure 3A) correlated with the Glisson's capsule invasion (P-value = 0.0122 and P-value = 0.0007, respectively).

**Figure 4:**
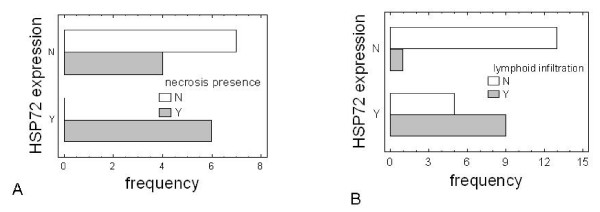
**HSP72 expression versus necrosis and lymphoid infiltration**. *Bar-chart *plots of HSP72 expression versus frequency of necrosis (A) and lymphoid infiltration (C).

pHH3 was associated with tumor greatest dimension and vascular invasion (P-values of 0.0264 and 0.025, respectively) (see Figure 3B and 3C). No significant correlation between HSP27 and the remaining variables was observed (Table 1). As to HSP72 expression, it was significantly (Figure 4A) associated only with tumor necrosis (P-value = 0.010) and lymphoid infiltration (P-value = 0.0023) (see Figure 4B).

**Figure 5:**
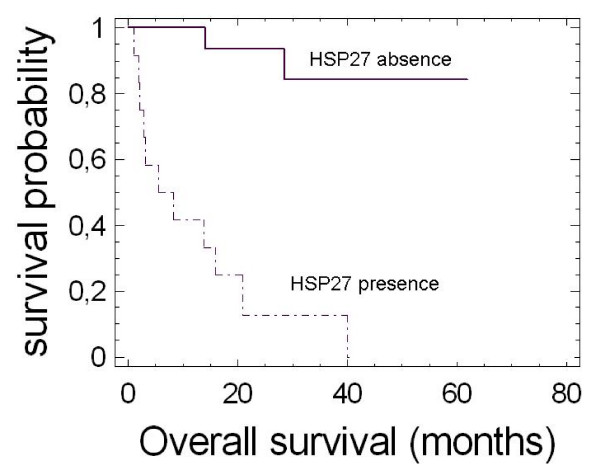
**Non parametric estimates of survival for HSP27 expression**. This figure shows the estimated survival probabilities based on the survival data of patients with presence or absence of HSP27 expression in their tumor samples.

Finally, we evaluated pHH3 and HSPs expression as predictors of survival. The percentage of pHH3 positive cells was significantly lower in survivors than in patients died for disease (2.71 ± 0.6% vs 4.98 ± 0.6%; P-value = 0.042). Kaplan-Maier analysis showed that patients with pHH3 index above cutpoint (2.4%, P-value = 0.034) had a shorter median overall survival (28.5 months) that did the patients with pHH3 index below cutpoint (> 50 months) (P-value = 0.025). The aggregate expression of HSPs was significantly associated (P-value = 0.0032) with a marked decrease of overall survival rates: the median survival for those patients with positivity for HSPs expression was 20.9 ± 8.3 months (with 13 deaths out of 21 patients) while was more than 57.5 months for those 10 patients without HSPs expression (only one patient died) with a power of 0.73. We then evaluated the relative contribution of the single expression of HSP27 or HSP72 to patients' overall survival. The presence of HSP72 did not affect patients'overall survival (53 vs 28.5 months P-value = 0.155, with a power of 0.51). However, due to the low value of power, we can not exclude that there is a statistically significant difference between the survival probabilities of the 2 groups. As shown in Figure 5, the HSP27 expression (with the concomitant expression of HSP72) was markedly associated (P-value = 0.0002) with a short overall survival (median survival: 11 months, with 11 deaths out of 14 patients) while in the absence of HSP27 the median survival was above 55 months with 3 deaths out of 17 patients. It is interesting to note that the presence of HSP27, in the absence of HSP72, determined the worst median survival (7 ± 3.2 months, with 4 deaths out of 6 patients).

In our population, the following factors, when evaluated by Cox regression univariate analysis (Table 2), significantly predicted the risk of death: vascular invasion, Glisson's capsule invasion, HSP27 expression, and stage. Owing to the stratification of the cohort, these last data have to be regarded for exploratory purpose. Since our cohort of patients was too small to detect independent prognostic factors, multivariable regression analysis was not performed.

**Table 2:** Cox univariate analysis

***Variable***	***Hazard Ratio***	***95%CI***	***P-Value***
Age	1.03	0.97 – 1.1	0.33
Gender	0.68	0.21 – 2.22	0.52
Tumor Border Growth^(1)^	3	0.85 – 10.6	0.088
Multiple nodules	0.923	0.31 – 2.75	0.89
**Vascular Inv.**	**3.18**	1.08 – 9.33	**0.036**
**Capsular Inv.**	**3.95**	1.20 – 12.9	**0.024**
Resected margins invasion	1.79	0.55 – 5.82	0.33
Tumor mass^(2)^	1.30	0.95 – 1.79	0.100
Number of resected segments	0.914	0.68 – 1.23	0.55
**HSP27**	**18.5**	3.83 – 89.6	**0.0003**
HSP72	1.99	0.66 – 5.94	0.22
**Stage**^(3)^	**7.315**	1.46 – 122.6	**0.032**

In bold are highlighted those variables with P-value < 0.05. ^(1)^Microscopic tumor border growth; ^(2)^Tumor greatest dimension; ^(3)^Coded as Early Stage (Stage I-II) or Late Stage (Stage III-IV).

## Discussion

Surgical resection is the mainstay of curative treatment for cholangiocarcinoma [1]. A neoadjuvant therapy or adjuvant therapy with radiation or chemotherapy has not been proven to prolong survival. Absence of lymph node involvement, negative tumour margins up to 1 cm, solitary lesions, and lack of microscopic vascular invasion correlate with improved survival. The most important prognostic factor after surgery is tumour-free surgical margins[3]. Even with careful selection and curative intent (R0 surgery), the 5-year survival ratio ranges from 30% to 40%[3,16].

In the last few years considerable efforts have been devoted to the search for markers of diagnosis and prognosis of IHCCA [17-19]. For instance, we have shown [13] that the combined expression of the Maspin and Bax proteins appears to be a predictor of survival in IHCCA likely influencing the susceptibility of tumor cholangiocytes to apoptosis. Altough human cholangiocarcinoma do not express the protooncogene Bcl-2, other antiapoptotic proteins of the Bcl family such as Mcl-1 and Bcl-xl are expressed[20]. Interestingly, Mcl-1 was found to mediate TRAIL resistance in cholangiocarcinoma cells by blocking the intrinsic pathway of apoptotic cell death[21]. In the present study we show that the immunohistochemical expression of HSP27 in tumor tissue significantly correlates with patients survival and might be regarded as a novel potential prognostic factor in IHCCA.

HSP27 and HSP72 are stress proteins inducible in response to a wide variety of insults[22]. Being powerful chaperones, their overexpression allows cells to survive otherwise lethal conditions[6,23]. This cytoprotective effect is related to their ability to disable apoptosis by acting at multiple control points of the apoptotic pathways[7]. It has been suggested that HSP27 and HSP72 partecipate in oncogenesis since their overexpression and the consequent inhibition of apoptosis can increase the tumorigenic potential of cancer cells[6].

HSP27 interacts with key apoptosis-associated proteins and inhibits cell death by a variety of mechanisms[24]. HSP27 can interfere with the intrinsic cell death pathway by preventing the formation of the apoptosome[6], and with the extrinsic apoptotic pathway by inhibiting Fas-induced signaling[25]. Furthermore, HSP27 has been shown to inhibit apoptosis by decreasing the reactive oxygen species subsequently to increase of glutathione and reduction of the toxic effect of oxidized proteins [26]. Recently, it has been characterized the tumorigenic role of Fas/FasL in cholangiocarcinoma and it has been suggested this pathway as a potential molecular target for therapeutics strategies to circumvent apoptosis-resistance of cholangiocarcinoma cells[27].

Higher than normal level of HSP27 expression was detected in breast, prostate, bladder[28], gastric[29], ovarian [30-32], and oral squamous carcinoma [33] cancers, as well as in Hodgkin's disease[34]. HSP27 level is generally low or absent within unstressed cells and increases during the stress response. However, the mechanisms that regulate HSP27 mRNA and protein levels have yet to be fully defined in cancer cells.

In our study the HSP27 expression in tumor tissue correlated with poor clinical outcome. Independently of the intensity of staining we also observed a significant association of HSP27 expression to pathological parameters related to tumor progression such as pHH3, gross tumor greatest dimension, vascular and capsular invasion[35,36]. It is interesting to note that this association applied only to the expression of HSP27 and not of HSP72. This observation is in agreement with recent reports indicating that the knock-down of HSP27 with siRNA or oligo anti-sense RNA in tumor cell lines induces apoptosis and enhances sensitivity to chemotherapic treatments[37,38]

Elevated expression of HSP72 either individually or in combination with other HSPs (HSP27 or HSP90) has been widely reported in several solid cancer[39], and various leukaemia [40-42]. The majority of the published results indicate that HSP72 expression correlated with poor prognosis and resistance to therapy. However, there are contradictory data of HSP72 in some tumors: for example, HSP72 expression correlated with poor prognosis in breast[43], endometrial[44,45], cervical/uterin cancer [39,46]. In contrast, high HSP72 expression correlated with good prognosis in esophageal[47], pancreatic and renal cancer[48]. Furthermore, HSP72 expression did not correlate with prognosis in ovarian, oral head and neck, oral squamous, gastric and prostate cancer.

Beside its antiapoptotic and tumorigenic role HSP72, when released from cells undergoing necrosis, might exert a proinflammatory effect owing to its interaction with receptors on inflammatory cells[49]. Under massive tumor necrosis the release of HSP72 can lead to specific lymphocyte-mediated anti-tumor immune response[50] that can induce tumor regression. Therefore, HSP72 can exert both positive and negative effects on tumor growth. Our results are in agreement with the aforementioned proinflammatory effect of HSP72, supporting the role of HSP72 in stimulating the immune response[51] and suggest a duality of function (antiapoptotic vs proinflammatory) in cancer development.

The present study suggests that HSP27 expression facilitates IHCCA progression likely by inhibiting apoptotic cell death, and the assessment of HSP27 expression by immunostaining in IHCCA might help to detect those patients with a high risk of death. Accordingly, univariate Cox regression analysis (see Table 2) shows a significant relation between the presence of HSP27 and probability to die.

The development of strategies targeting the modulation of HSP27 expression in IHCCA may allow control over tumor expansion and improve patients' survival. Downregulation of HSP27 gene expression by nucleotide-based therapies or increased phosphorylation (inactivation) of HSP27 protein by specific phosphatase inhibition could also restore tumor cell sensitivity towards apoptosis and chemosensitizes cells to chemotherapy [38].

Our study has the limitation of a low number of patients that is too small to draw valid conclusions or conduct further statistical analysis. However, our cohort represents a large single institution series given the rarity of this tumor. A multicentric and randomized trial will be required to validate the results performed in this explorative retrospective study. If confirmed in a large independent trial, the expression of HSP27 should help to discriminate IHCCA patients with a better prognosis. Moreover, preoperative informations retrieved from liver needle biopsies could provide a basis for selecting patients with a likely favorable course for surgery enrolling.

## Conclusion

Up to now there is no study of the relationship between HSPs expression and patient's prognosis in IHCCA. In this exploratory retrospective study the expression of HSP27 was found to be related to aggressive tumor behaviour. The expression of HSP27 significantly worsened the patients' median overall survival. The routinely histological detection of HSP27 expression may provide a reliable prognostic marker for IHCCA able to select those patients with a likely favorable course for surgery enrolling.

## Competing interests

The author(s) declare that they have no competing interests.

## Authors' contributions

AR conceived of the study, carried out the design and the immunohistochemical studies, performed the statistical analysis, and wrote the manuscript; PC supplied the samples, carried out the pathological staging, and reviewed the manuscript; GG and CL helped the pathologist and contributed to the results analysis; SD participated in the analysis of results and drafted the manuscript; MS reviewed the manuscript; PS supplied clinical data, reviewed the manuscript and acted as corresponding author; AFB conceived of the study, and helped in its design, coordination and critical review of the manuscript. All authors have read and approved the final manuscript.

## Pre-publication history

The pre-publication history for this paper can be accessed here:


